# Perinatal Referral and Admission Trends in Leeds Mother and Baby Unit 2023–2024

**DOI:** 10.1192/bjo.2025.10466

**Published:** 2025-06-20

**Authors:** Khadeeja Ansar, Gopinath Narayan, Jessica Hopwood, Nishi Shah

**Affiliations:** Leeds and York NHS Foundation Trust, Leeds, United Kingdom

## Abstract

**Aims:** Mother and Baby Units offer specialised treatment to women in the antenatal period from 32 weeks gestation to 12 months postpartum. All referrals are screened for admission suitability. Reasons to reject referrals include absence of serious mental illness and high risk of violence.

To analyse relationships between referrals received and accepted admissions regarding clinical and social variables, including deprivation levels, timing of referrals, diagnosis and ethnicity.

**Methods:** Retrospective data collection looking at all referrals to Yorkshire and Humber Mother & Baby Unit between 1 April 2023 and 31 March 2024. Total 129 referrals. Patient records were used for data collection.

**Results:** Deprivation decile: Most referrals were from the most deprived decile (35%). Least from the least deprived decile (3%). Of the most deprived decile referrals, 86% were accepted. All from least deprived decile were accepted.

**Ethnicity:** Most referrals were white British (71%), then Asian/Asian British (13%), then black/black British (6%). Least were ‘other ethnic group’ (4%). 6% had no ethnicity stated.

Of the referrals for white British ethnicity, 87% were accepted. For Asian/Asian British ethnicity, 94% were accepted. For black/black British ethnicity, 71% were accepted. For other ethnic group, 40% were accepted. With no ethnicity stated, 63% were accepted.

**Diagnosis:** Most referrals were for diagnosis of Psychotic Disorder (46%), followed by Mood Disorder (33%), Multiple (10%), Other (7%), and least for Anxiety Disorders (4%).

Of referrals for Psychotic Disorder, 88% were accepted. Mood Disorder, 90% were accepted. Multiple, 53% were accepted. Other, 66% were accepted. Anxiety Disorders, 80% were accepted.

**Time of Referral:** For point of referral in perinatal timeline, most referrals were between 2–12 weeks postpartum (36%), then 12+ weeks postpartum (32%), 0–2 weeks postpartum (20%), and least from pregnancy (12%).

At 2–12 weeks postpartum, 85% were accepted. At 12+ weeks postpartum, 88% were accepted. At 0–2 weeks postpartum, 96% were accepted. During pregnancy, 47% were accepted.

**Conclusion:** The data highlighted discrepancies in number of referrals received from different deprivation decile areas and ethnicities, significantly higher from more deprived areas and higher number of referrals for white British ethnicity patients. The acceptance proportion was higher in less deprived areas, this could be due to significant difference in number of referrals. The acceptance proportion for different ethnicities were fairly in the same range. Targeted interventions to promote awareness could improve equitable access.

